# Expanding the Clinical Spectrum Associated with the Recurrent Arg203Trp Variant in *PACS1*: An Italian Cohort Study

**DOI:** 10.3390/genes16020227

**Published:** 2025-02-16

**Authors:** Stefano Pagano, Diego Lopergolo, Alessandro De Falco, Camilla Meossi, Sara Satolli, Rosa Pasquariello, Rosanna Trovato, Alessandra Tessa, Claudia Casalini, Lucia Pfanner, Guja Astrea, Roberta Battini, Filippo M. Santorelli

**Affiliations:** 1 Molecular Medicine, IRCCS Stella Maris Foundation, 56128 Pisa, Italy; stefanop@hotmail.it (S.P.); ale.deltafi@gmail.com (A.D.F.); camilla.meossi@fsm.unipi.it (C.M.); sara.satolli@gmail.com (S.S.); rosy.tr@hotmail.it (R.T.); aletessa@gmail.com (A.T.); 2 UOC Neurologia e Malattie Neurometaboliche, Azienda Ospedaliero-Universitaria Senese, Policlinico Le Scotte, 53100 Siena, Italy; diego.lopergolo@unifi.it; 3 Department of Medicine, Surgery and Neurosciences, University of Siena, 53100 Siena, Italy; 4 Department of Developmental Neuroscience, IRCCS Stella Maris Foundation, 56128 Pisa, Italy; rosa.pasquariello@fsm.unipi.it (R.P.); claudia.casalini@fsm.unipi.it (C.C.); lucia.pfanner@fsm.unipi.it (L.P.); guja.astrea@fsm.unipi.it (G.A.); roberta.battini@fsm.unipi.it (R.B.); 5 Department of Clinical and Experimental Medicine, University of Pisa, 56124 Pisa, Italy

**Keywords:** Schuurs–Hoeijmakers syndrome, *PACS1*, intellectual disability, literature review, c.607C>T, red flags

## Abstract

**Background/Objectives**: Schuurs–Hoeijmakers syndrome (SHMS), also known as PACS1 neurodevelopmental disorder, is a rare condition characterized by intellectual disability, distinctive craniofacial abnormalities, and congenital malformations. SHMS has already been associated with variants in the *PACS1* gene in 63 patients. In this study, we describe 10 new Italian SHMS patients all harboring the common de novo p.(Arg203Trp) variant. **Methods**: The 10 patients we studied were evaluated by clinical geneticists and child neurologists and a detailed description of clinical features was recorded. Data were then coded using the Human Phenotype Ontology (HPO) terms. The recurrent p.(Arg203Trp) variant in PACS1 was identified by clinical exome sequencing or whole exome sequencing in trio using standard methodologies. To facilitate mutation identification, we designed a new PCR-RFLP strategy adopting the endonuclease *Dpn*II. **Results**: We define a detailed clinical phenotyping in patients with intellectual disability and facial characteristics (thick eyebrows, down-slanting palpebral fissures, ocular hypertelorism, low-set ears, a thin upper lip, and a wide mouth) that can help clinicians form a more efficient diagnosis of SHMS even through neuroimaging and neuropsychological evaluation. **Conclusions**: Our series of 10 newly affected Italian children highlights specific clinical features that may help clinicians recognize and better manage this syndrome, contributing to precision medicine approaches in medical genetics.

## 1. Introduction

Schuurs–Hoeijmakers syndrome (SHMS, MIM # 615009; ORPHA:329224), also known as *PACS1* neurodevelopmental disorder, is a rare disorder characterized by intellectual disability (ID), language difficulties, behavioral abnormalities, and autism spectrum disorder (ASD), associated with abnormal craniofacial features and congenital malformations. Feeding difficulty is common, with 25% of patients requiring a gastrostomy tube to maintain an adequate caloric intake. Other common features include constipation, partial and tonic seizures with early- or infantile-onset, congenital heart defects, short stature, and microcephaly. Hypotonia is reported in about one-third of individuals and seems to improve over time. Neuroimaging shows hypoplasia or partial agenesis of the cerebellar vermis in most children (about 65%). Partial and tonic seizures occur in approximately 50–60% [1].

SHMS is an autosomal dominant hereditary disease caused by pathogenic variants in the *PACS1* gene located in 11q13.1-q13.2. *PACS1* (Phosphofurin acidic cluster sorting protein 1) is a trans-Golgi-membrane traffic regulator that directs protein cargo and cranial neural crest cell migration. Its expression is upregulated during embryonic brain development, with low expression after birth [1,2]. The findings of experiments in zebrafish, documenting a role for the zf-pacs1 protein in cranial neural crest cell migration, might explain the characteristic facial features seen in patients [2].

**Figure 1:**
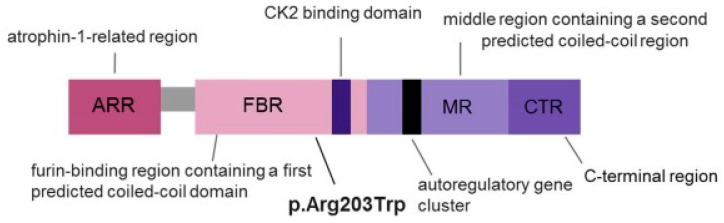
PACS1 (Phosphofurin acidic cluster sorting protein 1) domains and the location of the p.Arg203Trp variant.

*PACS1*-related disorders show low allelic heterogeneity, with most gene variants, including c.607C>T/p.(Arg203Trp) (Figure 1), showing a gain-of-function mechanism [3].

The identification of several individuals with the same de novo c.607C>T variant likely occurs during spermatogenesis, as seen in the case of *FGFR3* pathogenic variants described in achondroplasia. This could suggest a mutational hotspot in the gene [3].

The pertinent literature contains only a few cohort studies describing the spectrum of features seen in SHMS [3,4], and several single case reports, including two previously described Italian patients [5]. Here, we present a new cohort of Italian patients affected by SHMS, and outline features that may allow for a more precise definition of the syndrome.

## 2. Materials and Methods

We searched for Italian patients with a new diagnosis of SHMS, the recurrent de novo variant c.607C>T/p.(Arg203Trp), and availability of both parents for genetic tests to confirm the de novo status of the variant.

Ten Italian patients newly diagnosed with SHMS (4 males and 6 females, aged from 2.9 to 22.2 years) were referred to the Department of Developmental Neuroscience at the IRCCS Fondazione Stella Maris over the past three years. All underwent clinical neuropsychological evaluation to establish their cognitive and language abilities and emotional-behavioral profiles. Given the severity of their clinical profiles, not all of the patients were evaluated with structured tests; in some cases, qualitative evaluation was performed, aimed at interpreting patients’ functioning with reference to Piaget’s stages of development [6]. Testable patients were administered age-appropriate versions of psychometric intellectual development scales, namely the Italian versions of the Bayley Scales of Infant and Toddler Development [7], the Griffiths Mental Developmental Scales [8], and the Wechsler Intelligence Scales in the different versions (for children/adolescents and adults) [9]. In addition, an adaptive behavior questionnaire (Vineland-II Adaptive Behavior Scales [10]) was administered to caregivers. Language evaluation included assessment of productive [11] and receptive vocabulary [12], sentence comprehension, and sentence repetition tests [13]. The most severe and/or non-testable patients were evaluated by means of a parents’ questionnaire [14]. The patients’ clinical profiles are reported in Table 1.

The 10 patients we studied were evaluated by clinical geneticists as they showed an association between ID and distinctive craniofacial features, highly suggestive of those reported in SHMS children (Table 2). Information was collected on pregnancy, peripartum, neurodevelopmental milestones, and medical examinations performed (particularly EEG and MRI). During the physical examination, height, weight, and head circumference were measured and dysmorphic features were assessed. Data were then coded using the Human Phenotype Ontology (HPO) terms listed in Appendix A.

Clinical and genetic analyses were conducted in accordance with the principles of the Declaration of Helsinki and its 2024 implementation. Having obtained parental informed consent, genomic DNA was purified from peripheral blood samples. None of the parents were consanguineous, and the data on the perinatal period contained nothing of note in any patient.

The patients were diagnosed through the finding of the recurrent de novo variant c.607C>T/p.(Arg203Trp) by clinical exome sequencing or whole exome sequencing in trio using standard methodologies Whole-exome sequencing (WES) was performed, and DNA libraries were prepared for each DNA sample using the SureSelect Human All Exon V7 system (Agilent Technologies, Santa Clara, CA, USA) in accordance with the manufacturer’s instructions. About 98% coverage of the mappable target > 20× was taken as the quality criterion. Exome data were analyzed using the enGenome-eVai (https://evai.engenome.com; v.3.1) and Integrative Genomic Viewer (IGV; http://igv.org/, accessed on 10 February 2024) tools. Databases of pathogenic variants, such as VarSome (https://varsome.com/) and Franklin Genoox (https://franklin.genoox.com/), were also reviewed to assess allele frequencies. The variant was reviewed, classified, and interpreted according to the American College of Medical Genetics and Genomics guidelines [15].

**Figure 2:**
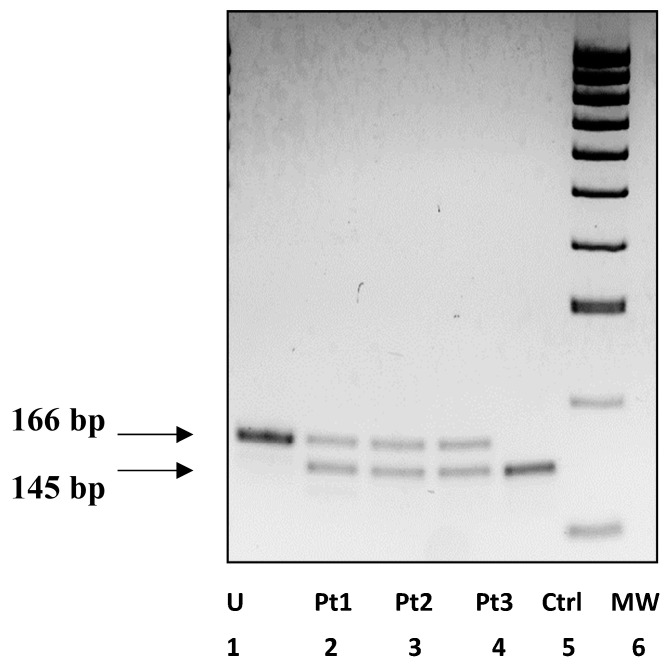
PCR-RFLP analysis of the c.607C>T variant in *PACS1*. Lane 1: U = uncut; lanes 2–3–4: patients Pt1–Pt3 harboring the heterozygous c.607C>T/p.(Arg203Trp) mutation; lane 5: Ctrl, negative control; lane 6: MW, 100-bp DNA molecular weight marker. The amplified PCR product was cleaved with the endonuclease *Dpn*II. Cleavage in the wild-type allele resulted in two fragments of 145 and 21 bp (undetectable), respectively, whereas the presence of the c.607C>T variant abolished the single site of cleavage, resulting in an uncut 166-bp fragment. Heterozygous patients showed 166-, 145-, and 21-bp (undetectable) fragments.

To facilitate mutation identification, we designed a polymerase chain reaction–restriction fragment length polymorphism (PCR-RFLP) strategy to rapidly detect the common pathogenic c.607C>T variant in *PACS1* (NM_018026) by removing the single site of cleavage for the endonuclease *Dpn*II (/GATC) in a 167-base pair (bp) amplicon obtained using mismatched forward primer 5′-CAAAGGAGAAAACGTTACAAGGAT-3′ (the mismatched nucleotide is underlined), and reverse primer 5′-CATCTGGAAGGCTCAACTGGC-3′. The amplified PCR product was cleaved with the endonuclease *Dpn*II and electrophoresized on a 2.5% agarose gel resulting in 145- and 21-bp fragments. The presence of the c.607C>T variant removes the single site of cleavage (Figure 2), resulting in 166-, 145-, and 21-bp fragments in the heterozygous index cases.

## 3. Results

We collected index cases in 10 unrelated kindreds, all harboring the most common pathogenic variant in *PACS1* [(NM_018026.4): c.607C>T/p.(Arg203Trp), chr11-65978677 C>T]. Segregation analysis showed that this variant arose de novo in all the cases. Mosaicism in the parental DNA was excluded by exploring the sequence traces using the Integrative Genomics Viewer (https://igv.org/doc/desktop/; accessed on 10 February 2024).

Evaluation of the neuropsychological profile revealed that all the patients exhibited ID and language impairment, in most cases moderate or severe; in younger patients, language was absent (40%), confirming that language development is more severely impaired than motor milestones in this disease. All the patients presented behavioral, emotional, and socio-relational deficits: autistic traits were detectable in 50% of cases, and emotional–behavioral dysregulation was found in 50% of them.

Physically, all the patients exhibited distinctive craniofacial features, including down-slanting palpebral fissures, thick eyebrows, long eyelashes, ocular hypertelorism, low-set ears, a bulbous nose, a broad nasal bridge, a wide mouth, and a thin upper lip.

**Figure 3:**
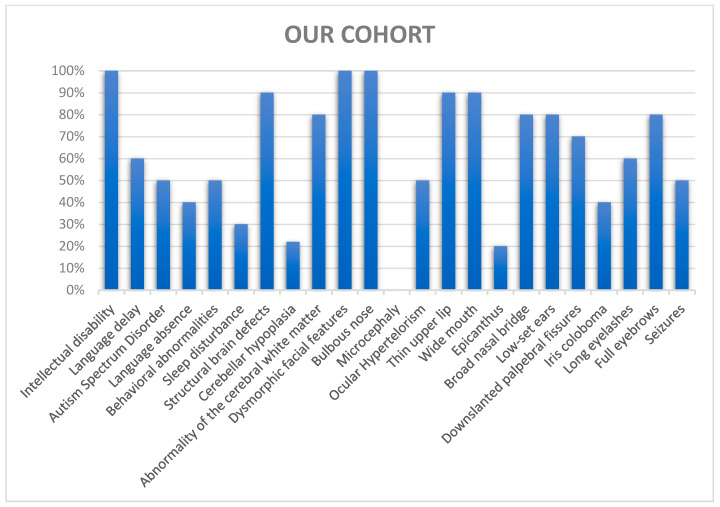
Chart of the clinical features in our cohort.

**Figure 4:**
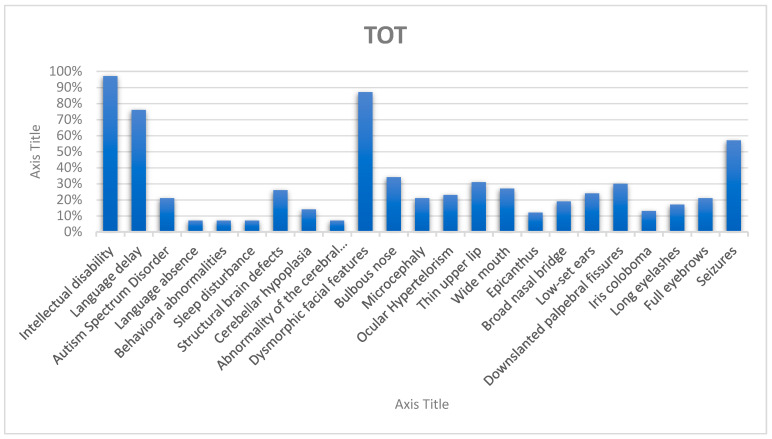
Chart of clinical features in all the patients considered.

We focused on neurodevelopmental, neuroradiological, and dysmorphic characteristics to perform phenotype–genotype correlation in our cohort and compare our findings with literature data on SHMS (Table 2), (Figure 3 and Figure 4).

**Figure 5:**
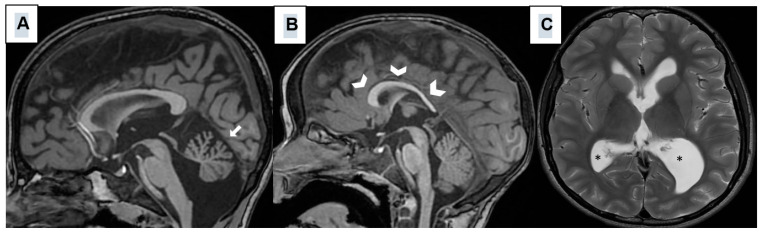
Sagittal T1-weighted (**A**,**B**) and axial T2-weighted (**C**) MRI images obtained from three different SHMS patients and showing hypoplasia/atrophy of the cerebellar vermis (white arrow); (**A**), thin and dysmorphic corpus callosum (thick arrowhead) (**B**), enlarged and asymmetric lateral ventricles (*) (**C**).

All the patients underwent brain MRI, which showed widespread malformations of the cerebellum, corpus callosum, and lateral ventricles. Figure 5 shows representative scans.

## 4. Discussion

SHMS, initially described in 2012, is an uncommon cause of ID. Its prevalence rate is probably underestimated due to often non-specific clinical features. Of the more than 150 known cases, approximately 63 have thus far been documented molecularly in the literature [16,17]. Patients with SHMS may be more easily recognized when ID and language abnormalities are associated with certain facial features: down-slanting palpebral fissures, bulbous nose, broad nasal bridge, ocular hypertelorism, low-set ears, wide mouth, thin upper lip, thick eyebrows, and long eyelashes.

Follow-up in SHMS currently consists of monitoring developmental progression, growth parameters, and behavioral parameters, as well as cardiac septal defects and renal malformation when present. It is also important to monitor patients for the onset of constipation, respiratory insufficiency, and seizures. In the absence of disease-modifying therapies, there are ongoing efforts to develop an anti-sense oligonucleotide therapy that would work by binding the mutant RNA and blocking the negative effect of the aberrant protein [18].

Here, we reviewed the clinical and molecular features of 10 new Italian SHMS patients. Compared with SHMS patients reported in the literature, our cohort showed a higher prevalence of speech abnormalities (40% vs. the 7% reported by reported by Tenorio-Castaño et al.). Behavioral issues and diagnosed ASD were each found in half of the sample, with frequencies much higher than those given in other studies or case reports (10% and 17%, respectively). In this case, the difference could be explained by the methodology used in the dysmorphological physical examination.

**Figure 6:**
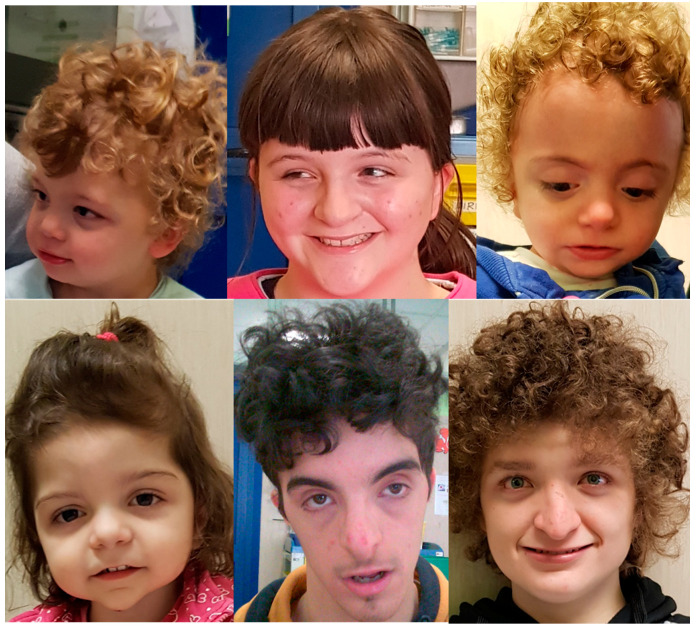
Dysmorphic facial features in six of our patients: down-slanting palpebral fissures, thick eyebrows, ocular hypertelorism, low-set ears, bulbous nose, broad nasal bridge, wide mouth, and thin upper lip.

Regarding the presence of dysmorphic features, a significant percentage of our patients presented with iris coloboma (40% versus 9% in the Tenorio-Castaño et al. cohort). Conversely, none of our patients exhibited microcephaly (compared with 21% in in Tenorio-Castaño et al. cohort). In this case, the differences could be explained by the methodologies. To help physicians recognize the syndrome through its clinical features (Figure 3), we recommend focusing primarily on the specific association between ID and the presence of facial dysmorphisms, particularly down-slanting palpebral fissures, thick eyebrows, low-set ears, bulbous nasal tip, thin upper lip, and wide mouth (Figure 6). It is always advisable to check for the presence of iris coloboma too. In terms of neurodevelopmental management, we recommend longitudinal neuropsychological evaluation to assess cognitive and language abilities and emotional–behavioral profiles. A further MRI evaluation is suggested if behavioral symptoms worsen. When SHMS is strongly suspected, a direct test for the pathogenic c.607C>T variant should be proposed, perhaps with the use of a rapid PCR-RFLP strategy.

## 5. Conclusions

SHMS, due to mutations in *PACS1*, is a rare cause of ID, which can be identified during clinical examinations also on the basis of certain facial characteristics, such as thick eyebrows, down-slanting palpebral fissures, ocular hypertelorism, low-set ears, a thin upper lip, and a wide mouth. Our series of 10 newly affected Italian children highlights specific clinical features that may help clinicians recognize and better manage this syndrome. This work contributes to precision medicine by enabling earlier and more accurate diagnoses of rare intellectual disability syndromes; the genetic diagnosis not only facilitates targeted interventions but also informs surveillance and counseling, particularly for conditions with overlapping phenotypes.

## Figures and Tables

**Table 1:** Patients’ clinical profiles evaluated with reference to Piaget’s criteria.

Patient	Sex	Age	Intellectual Disability	Language	Behavioral and Socio-Relational Abnormalities
C.V.	F	2.1	Moderate (acquisition of sensorimotor skills, deficit in adaptive behavior)	Severely impaired (non-verbal)	Emotional–behavioral dysregulation and stereotyped movements
T.B.	F	2.7	Moderate (acquisition of sensorimotor skills, deficit in adaptive behavior)	Severely impaired (non-verbal)	Stereotyped movements
Q.F.	M	2.9	Severe (acquisition of sensorimotor skills, deficit in adaptive behavior)	Severely impaired (non-verbal)	Autistic features
R.E.	M	3.7	Moderate (acquisition of sensorimotor skills, severe deficit in adaptive behavior)	Severely impaired (non-verbal)	Autistic features and emotional–behavioral dysregulation
M.I.	F	12.10	Severe (acquisition of early visual/graphic representation skills, deficit in adaptive behavior)	Severely impaired (stereotyped sentences)	Autistic features
C.G.	F	15.1	Moderate (acquisition of early preoperational skills, severe deficit in adaptive behavior)	Moderately impaired (can pronounce simple and complex sentences)	Emotional–behavioral dysregulation
S.C.	F	16	Moderate (acquisition of preoperational skills, severe deficit in adaptive behavior)	Mildly impaired (can pronounce simple and complex sentences)	Emotional–behavioral inhibition
D.D.V.	M	17.5	Severe (acquisition of early visual/graphic representation skills and severe deficit in adaptive behavior)	Severely impaired (stereotyped sentences)	Autistic features
S.N.	M	19.1	Mild (acquisition of formal operational skills, mild deficit in adaptive behavior)	Mildly impaired (can pronounce simple and complex sentences)	Autistic features
S.G.	F	22.2	Severe (acquisition of early visual/graphic representation skills, severe deficit in adaptive behavior)	Severely impaired (presents some vowel productions and few words)	Emotional–behavioral dysregulation

**Table 2:** Summary of a suggestive phenotype of a SHMS patient.

Category	Features
**Craniofacial Features**	- Thick eyebrows - Down-slanting palpebral fissures - Ocular hypertelorism - Low-set ears - Wide mouth with a thin upper lip
**Neurodevelopmental Findings**	- Global developmental delay - Intellectual disability - Language delay or Language absence - Behavioral abnormalities
**Neuroimaging Abnormalities**	- Cerebellar Hypoplasia (hypoplasia of the cerebellar vermis) - Mild ventriculomegaly - Hypoplastic corpus callosum - Abnormality of cerebral white matter

## Data Availability

The original contributions presented in this study are included in the article. Further inquiries can be directed to the corresponding author.

## Abbreviations

The following abbreviations are used in this manuscript:

SHMS	Schuurs–Hoeijkakers syndrome
ID	intellectual disability
ASD	autism spectrum disorder
HPO	human phenotype ontology
PCR-RFLP	polymerase chain reaction–restriction fragment length polymorphism
bp	base pair

## Appendix A

**Table A1:** Traits (HPO) of the patients. The overall Italian cohort includes patients described in the present work and two previously described case reports [5]. Tenoro-Castaño et al. [4] include the most recent systematic reviews of SHMS patients.

Trait (HPO)	Present Cohort	Overall Italian Cohort	Tenorio-Castaño et al., 2021 [4]
**Neurodevelopmental disorders**			
Intellectual disability (HP:0001249)	10/10 100%	12/12 100%	56/58 97%
Language delay (HP:0000750)	6/10 60%	8/12 66%	42/55 76%
Language absence (HP:0001344)	4/10 40%	4/12 33%	4/56 7%
Autism spectrum disorder (HP:0000729)	5/10 50%	5/12 41%	12/56 21%
Behavioral and/or emotional abnormalities (HP:0000708)	5/10 50%	5/12 41%	4/56 7%
Sleep disturbance (HP:0002360)	3/10 30%	3/12 25%	4/56 7%
**Brain abnormalities**			
Structural brain defects (HP:0012443)	9/9 100%	10/11 90%	13/49 26%
Cerebellar hypoplasia (HP:0001321)	2/9 22%	2/11 18%	6/43 14%
Abnormalities of the cerebral white matter (HP:0002500)	8/10 80%	8/12 66%	3/43 7%
**Craniofacial features**			
Dysmorphic facial features (HP:0001999)	10/10 100%	12/12 100%	49/60 82%
Bulbous nose (HP:0000414)	10/10 100%	12/12 100%	13/61 21%
Microcephaly (HP:0000252)	0/10 0%	0/12 0%	13/61 21%
Ocular hypertelorism (HP:0000316)	5/10 50%	7/12 58%	13/61 21%
Thin upper lip (HP:0000219)	9/10 90%	11/12 91,6%	11/61 18%
Wide mouth (HP:0000154)	9/10 90%	11/12 91,6%	8/61 13%
Epicanthus (HP:0000286)	2/10 20%	4/12 33%	6/61 10%
Broad nasal bridge (HP:0012811)	8/10 80%	10/12 83%	6/61 10%
Low-set ears (HP:0000369)	8/10 80%	8/12 66%	9/61 15%
Down-slanted palpebral fissures (HP:0000494)	7/10 70%	9/12 75%	14/61 23%
Iris coloboma (HP:0000612)	4/10 40%	4/12 33%	5/57 9%
Long eyelashes (HP:0000527)	6/10 60%	6/12 50%	7/61 12%
Full eyebrows (HP:0004523)	8/10 80%	9/12 75%	7/61 12%
**Other**			
Seizures (HP:0001250)	5/10 50%	6/12 50%	33/58 57%
